# Predictive modeling of anti-malarial molecules inhibiting apicoplast formation

**DOI:** 10.1186/1471-2105-14-55

**Published:** 2013-02-15

**Authors:** Salma Jamal, Vinita Periwal, Open Source Drug Discovery Consortium, Vinod Scaria

**Affiliations:** 1 CSIR Open Source Drug Discovery Unit, Anusandhan Bhavan, Delhi 110001, India; 2 GN Ramachandran Knowledge Centre for Genome Informatics, CSIR Institute of Genomics and Integrative Biology, Mall Road, Delhi 110007, India

## Abstract

**Background:**

Malaria is a major healthcare problem worldwide resulting in an estimated 0.65 million deaths every year. It is caused by the members of the parasite genus *Plasmodium*. The current therapeutic options for malaria are limited to a few classes of molecules, and are fast shrinking due to the emergence of widespread resistance to drugs in the pathogen. The recent availability of high-throughput phenotypic screen datasets for antimalarial activity offers a possibility to create computational models for bioactivity based on chemical descriptors of molecules with potential to accelerate drug discovery for malaria.

**Results:**

In the present study, we have used high-throughput screen datasets for the discovery of apicoplast inhibitors of the malarial pathogen as assayed from the delayed death response. We employed machine learning approach and developed computational predictive models to predict the biological activity of new antimalarial compounds. The molecules were further evaluated for common substructures using a Maximum Common Substructure (MCS) based approach.

**Conclusions:**

We created computational models using state-of-the-art machine learning algorithms. The models were evaluated based on multiple statistical criteria. We found Random Forest based approach provides for better accuracy as assessed from ROC curve analysis. We further evaluated the active molecules using a substructure based approach to identify common substructures enriched in the active set. We argue that the computational models generated could be effectively used to screen large molecular datasets to prioritize them for phenotypic screens, drastically reducing cost while improving the hit rate.

## Background

Malaria is a major health problem across the world, more so in the tropics and especially in developing nations [1]. According to the recent World Malaria Report, released by the World Health Organization (WHO) in 2011, there were about 216 million cases of malaria across the globe and 0.65 million deaths in 2010; with highest mortality found within children living in Africa [2,3]. Malaria is a mosquito-borne disease and is caused by protozoan parasites belonging to the genus *Plasmodium*. *P. falciparum, P. vivax, P. ovale* and *P. malariae* are the four species of the parasite which are routinely implicated as the causative agents in humans, with *P. falciparum* being the most commonly encountered and deadliest amongst them all and associated with 90% of the fatalities in Africa [4,5]. Endemic to the tropical and subtropical regions of Africa, Asia, South and Central America where hot and humid climatic conditions prevail, malaria has been indicated as a major constraint to economic development [6-8].

One of the major roadblocks in the adequate control of malaria has been the limited therapeutic options available for its treatment. The current commonly used classes of drugs are limited to aminoquinolines and their derivatives such as arylamino alcohols, methanols, biguanides, diaminopyrimidines and antimalarial endoperoxidases. Chloroquine and primaquine have been extensively used for the treatment and prophylaxis of malaria [9,10]. However, widespread drug resistance to available therapeutic agents and the emergence of multi-drug resistant strains has resulted in limited treatment options [11-14]. The current pipeline for drug discovery of anti-malarials is also limited, with just 13 products in clinical trials and 8 in preclinical stages of development [15]. Large scale collaborative initiatives have made it possible to assemble large datasets of chemical structure information online [16]. This has been complemented by the annotation of biological activities of these molecules. Many of the biological activities have been derived by high-throughput bioassays made possible by recent advances in automation of these assays. The availability of chemical structure and bio-activity information in standardized forms provide immense opportunities for creating predictive computational models to understand the correlation between chemical properties and their activities and also opens up the possibility to create predictive computational models for bio-activities [17,18]. These predictive models make it possible to computationally screen large molecular datasets thereby offering a possibility to improve the hit-rate and thereby reduce the overall costs of drug discovery. We have also previously successfully generated such predictive models for anti-tubercular molecules [19,20] and for small molecule modulators of miRNA [21].

In the present study, we applied the machine learning technique to create classification models from high-throughput screens of anti-malarial agents that inhibit the development of the apicoplast in the malaria parasite, *P. falciparum*. In addition, we used a Maximum Common Substructure (MCS) based approach to identify substructures enriched in the bioactive molecules. Our result suggests that efficient and accurate computational predictive models could be built to screen large datasets *in silico* and could be potentially used to prioritize molecules for high-throughput screens.

## Results and discussion

### Descriptor generation and model construction

Initially, a total of 179 2D molecular descriptors were generated for the active and inactive datasets downloaded from PubChem. After data processing, as explained in methods section, the number of descriptors was reduced to 154 (Additional file 1), since not many descriptors were removed after data processing, we assumed the compounds to be structurally diverse. As the dataset used in the study was large, the heap-size in Weka was increased to 4 GB to handle out-of-memory exception. The initial experiments were done using standard base classifiers; however, to reduce the rate of False Negatives, cost sensitivity was introduced in classifiers using the meta-learners. Misclassification cost was set for False Negatives and was incremented so as to stay around the upper limit of False Positives (i.e., 20%). As expected, introducing cost for each of the classifier resulted in an increase in the number of True Positives and decrease in the number of False Negatives thereby increasing the robustness of the model. The final misclassification cost used for each classifier is presented in Table 1. The Naive Bayes classifier required the smallest misclassification cost setting and was also the fastest in building the model.

**Table 1:** Classification results

Classifier^*^	**TP rate**	**FP Rate**	**TN rate**	**FN rate**	**ROC area**	**Accuracy (%)**	**BCR**^**#**^	**Cost**
**CSC NB**	41.8	21.4	78.6	58.2	65.1	74.81	59.5	2
**CSC RF**	51	20.9	79.1	49	70.8	76.27	64	40
**MetaCost J48**	44.6	21.1	78.9	55.4	62.3	75.38	61	9

^*^CSC denotes *CostSensitiveClassifier,*^#^Balanced Classification Rate.

### Model evaluation

**Figure 1:**
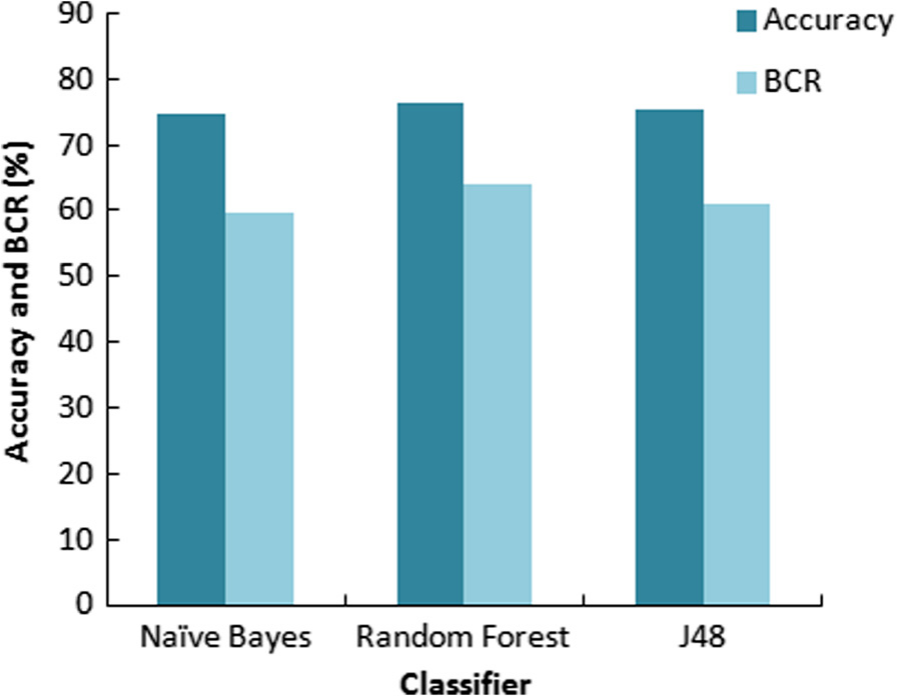
Comparison of accuracy and balanced classification rate.

**Figure 2:**
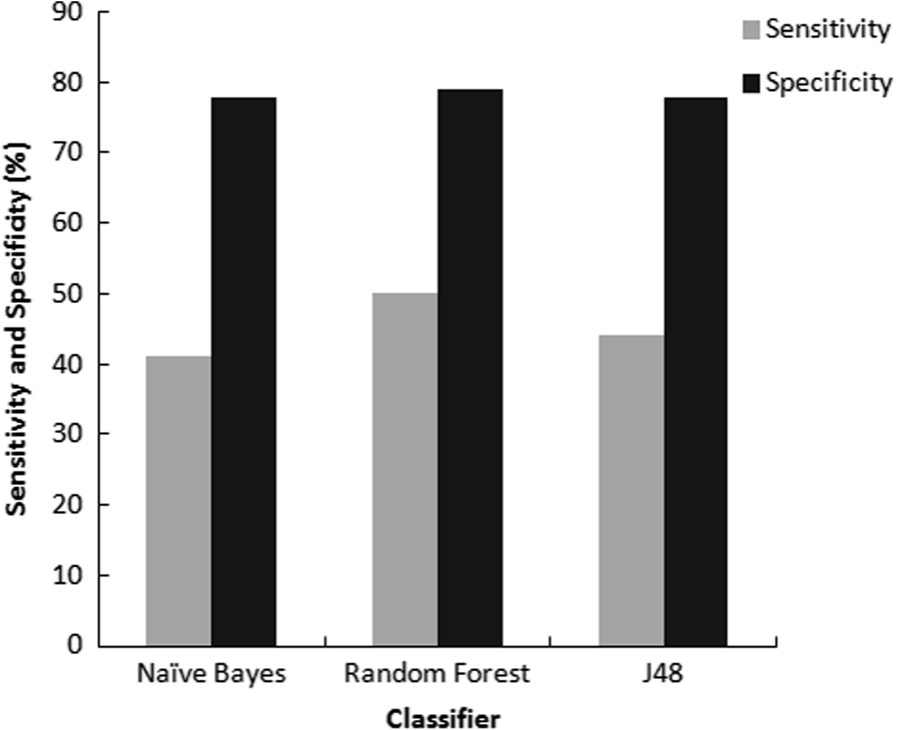
Plot of sensitivity and specificity.

A number of models were trained using 5-fold cross validation on the training dataset using different misclassification cost settings for False Negatives until cost optimized models were obtained. The best model for each classifier NB, RF and J48, was chosen based on their performance evaluated using different statistical measures (Table 1). All statistical results reported in Table 1 are based on independent test set and not on the training set. The overall efficiency of a classifier in generating the models was judged from the accuracy. The accuracy for all the models came out to be around 75% (Figure 1). Sensitivity and specificity plots were used for identifying the best models for each dataset for evaluating the effectiveness of the classifier in correctly identifying positive and negative labelled instances (Figure 2). The specificity for all the models was approximately 80% and the sensitivity ranged from 40-50% with RF being the most sensitive classifier for the dataset and NB the least sensitive.

**Figure 3:**
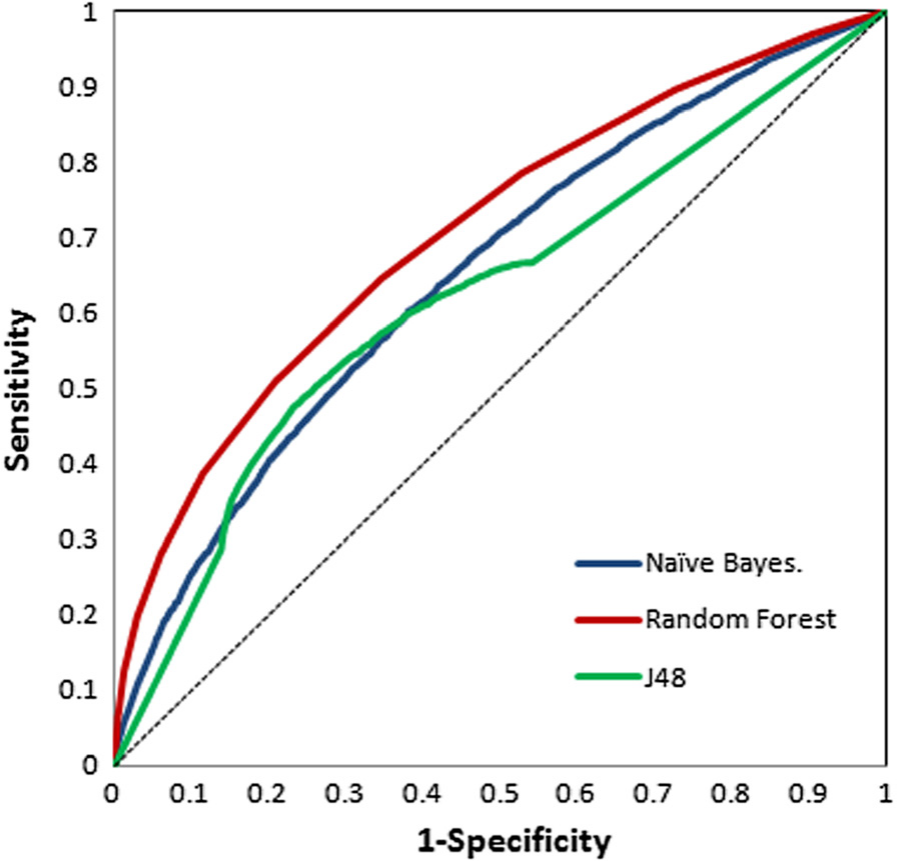
ROC plot depicting significant AUC curve values for Random Forest, Naïve Bayes and J48.

Since our dataset was highly imbalanced, accuracy alone cannot be used as a reliable statistical measure for assessment of the classifiers performance. In addition to this, other performance measures were employed to check the robustness of the model which included the BCR rate and ROC curve analysis. The balanced accuracy values turned out to be satisfactory for all the models with best for Random forest (Table 1), being more accurate than Naive Bayes and J48. ROC curve analysis has been widely accepted as one of the most reliable approach for quick performance assessment of virtual screening approaches therefore, it has been widely deployed in evaluating the discriminatory power of virtual screens. All the models had significant area under curve (AUC) obtained from ROC plot of the three classifiers depicted in the Figure 3. Random forest on the whole establishes to be the best classifier followed by NB and J48 producing a significant AUC of 70% as compared to NB (65%) and J48 (62%).

### Evaluation of substructures

**Figure 4:**
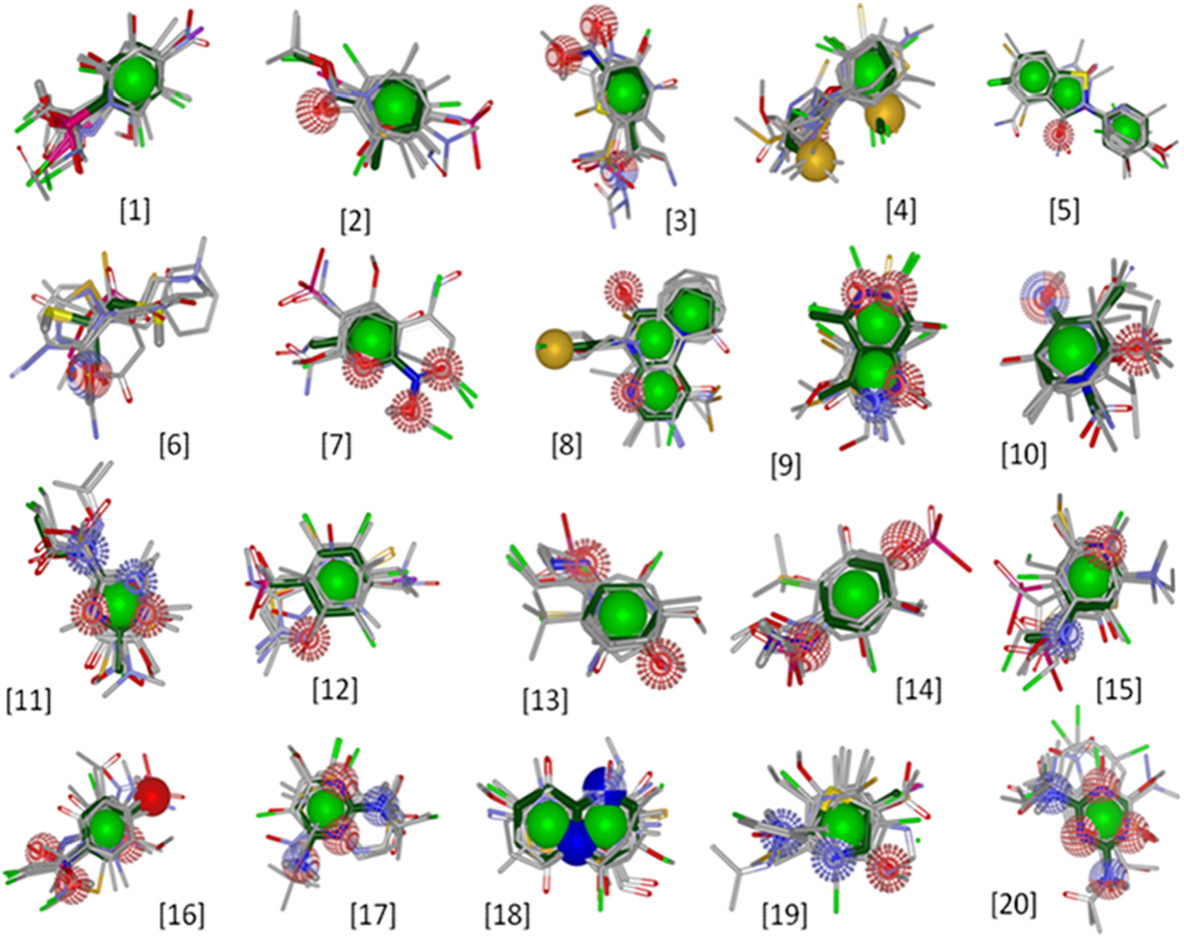
**Molecular overlay.** Alignment of 20 enriched scaffolds (dark green) with top 20 compounds of active dataset. Ranking was obtained from their Tanimoto similarity and overlap with the reference scaffold.

For identification of potentially enriched substructures in the bioactive molecules, the active dataset containing 22,335 compounds were clustered using *LibMCS* algorithm. All the ~22 k compounds were clustered into 1,842 scaffolds spread over 5 hierarchical levels. Only top level clusters were selected for further analysis. There were a total of 295 clusters at level 5 which included 80 singletons. As our aim was to identify potentially enriched substructures, all singletons were removed and only 215 scaffolds were taken up for further analysis. The number of occurrences of each of the 225 scaffolds in the active and the inactive datasets was determined. Chi-square test and p-value were used to determine the significance of enrichment (Table 2). 20 scaffolds had p-value less than 0.01 and an enrichment factor > 2. In order to assess the structural similarity of the scaffolds with the active molecules, the final 20 scaffolds were aligned against the active molecule dataset. Figure 4 represents an alignment generated with the top 20 compounds of the active set as determined from Tanimoto similarity and overlap between query scaffold and active molecules.

**Table 2:** Significantly enriched scaffolds in the active dataset

**Scaffold No.**	**Structure**	**Actives**	**Inactives**	**Chi-square**	**p-value**	**Enrichment Factor**
Scaffold 1		21	4	8.52	3.51E-03	46.39
Scaffold 2		4	1	26.70	2.37E-07	35.34
Scaffold 3		25	17	23.61	1.18E-06	12.99
Scaffold 4		7	7	12.78	3.49E-04	8.83
Scaffold 5		2	2	11.75	6.07E-04	8.83
Scaffold 6		2	2	6.95	8.38E-03	8.83
Scaffold 7		95	166	196.90	9.88E-45	5.05
Scaffold 8		6	11	17.67	2.62E-05	4.82
Scaffold 9		43	80	82.84	8.89E-20	4.74
Scaffold 10		24	54	36.26	1.72E-09	3.92
Scaffold 11		4	9	6.04	1.40E-02	3.92
Scaffold 12		22	54	29.36	5.99E-08	3.60
Scaffold 13		201	570	241.30	1.58E-48	3.11
Scaffold 14		10	29	10.48	1.20E-03	3.04
Scaffold 15		134	392	135.32	2.81E-31	3.02
Scaffold 16		9	31	6.66	9.83E-03	2.56
Scaffold 17		48	181	29.25	6.36E-08	2.34
Scaffold 18		126	488	72.29	1.85E-17	2.28
Scaffold 19		41	178	17.57	2.77E-05	2.03
Scaffold 20		164	722	67.82	1.79E-16	2.00

## Conclusions

Malaria is a neglected tropical disease. Widespread drug-resistance to commonly used anti-malarials which has limited the therapeutic options available has warranted the need to search for novel molecules with anti-malarial activity. The availability of high-throughput chemical screens in the public domain provides an excellent opportunity to create predictive computational models to prioritize molecules using a virtual screening approach. Such an approach therefore will, not only serve to aid the rapid screening of compounds but also subsequently enhance the identification of true hits and thereby would lead to reduced cost of carrying out biological screens. Our analysis shows that a systematically designed computational model for activity based on chemical descriptors could be potentially used for virtual-screening. The work encompasses a machine learning based framework to build *in silico* predictive models based on datasets from high-throughput screens for apicoplast inhibitors of the malaria parasite. Comparative analysis of various classifiers revealed that Random Forest performed better than both Naive Bayes and J48. The study was extended further to explore potentially enriched substructures in bioactive molecules, which resulted in the identification of 20 significantly enriched scaffolds. Predictive models in conjunction with the enriched scaffold information can be potentially used as a molecular filtering criterion for prioritizing molecules for biological screens for anti-malarial activity.

## Methods

### Source of bioassay data

The cell based assay used in the current study [AID: 504834] consists of antimalarial compounds and was obtained from PubChem database maintained by National Center for Biotechnology Information (NCBI) [16]. Briefly, the bioassay contained compounds which have the potential to inhibit apicoplast formation in *Plasmodium*. The assay was based on a Luciferase reporter assay and the compounds that cause inhibition of apicoplast formation was assayed by a delayed death response at 96 hours. The dataset AID: 504834 contained a total of 323,201 tested compounds. Compounds having a PubChem activity score between 40 and 100 were considered as active (N = 22,335), and all compounds with a score of 0 were considered as inactive (N = 197,373). Besides the active and inactive set of compounds, the assay depositor also reported two other sets consisting of inconclusive and unspecified compounds which were excluded from our study because of the un-certainty in their bioactivities. The compounds from the active and inactive datasets were downloaded in Structural Data Format (SDF).

### Descriptor generation and data pre-processing

2D molecular descriptors were generated for the molecules in the active and inactive datasets using PowerMV [22]. PowerMV is popular software used for descriptor generation statistical analysis and molecular similarity search and extensively used in the field. The datasets contained large number of chemical compounds which could not be processed in one single run, so they were initially split into smaller SDF files using SplitSDFiles Perl script available from Mayachem tools [23]. A total of 179 descriptors were generated using PowerMV. Among the descriptors generated, 147 belonged to pharmacophore fingerprints while 24 belonged to weighted burden numbers and 8 were property descriptors (Additional file 1). For the bit string descriptors, the attributes having only one value (all 0’s or all 1’s) throughout the dataset were filtered out to reduce the dimensionality of the dataset. Using a custom script, the dataset was split randomly into 80% train-cum-validation set and a 20% independent test set. A 5-fold cross validation was employed for training and validation set.

### Cost sensitive classifiers

Machine Learning (ML) is a scientific discipline that deals with the generation of predictive models based on known properties learned from training datasets. In this particular scenario, ML was employed to create binary classifiers for the molecules based on their bio-activity viz., actives and inactives. One of the issues to keep in consideration while using standard classifiers for model building is the imbalanced nature of the dataset, i.e. the class imbalance problem. Class imbalance arises from the fact that in most of the high-throughput unbiased screens, the numbers of inactive molecules exceeds far beyond the number of actives, the minority ratio being 11% in our study. Standard classifiers that use equal weighting for all the classes are incapable to handle such highly imbalanced data and tends to assume that all misclassification errors cost equally. One of the alternatives for this is to use cost sensitive classifiers in which misclassification costs are used [24]. We applied Weka (Waikato Environment for Knowledge Analysis) [25], a popular suite of machine learning algorithms in our study. Weka supports algorithms for data pre-processing, analysis, classification, clustering, feature selection techniques and visualization tools. Weka introduces cost sensitivity in the base classifiers by means of a confusion matrix, which for a binary classification scheme consists of four sections: True Positives (TP) for actives correctly classified as actives; False Positives (FP) for inactives incorrectly classified as actives; True Negatives (TN) in which inactives correctly classified as inactives and False Negatives (FN) for active compounds incorrectly classified as inactive. As False Negatives are considered more important in an experiment for compound selection, we set misclassification cost for False Negatives to lessen the False Negatives number at the cost of increasing the False Positives. However, increasing the cost for False Negatives will increase both the False Positives and True Positives. Therefore we set an empirical upper limit of 20% on the False Positive rate. Setting of the misclassification cost is always arbitrary and no general rule exists for it. It is more or less dependent on the base classifier used.

### Classification algorithms

Machine learning encompasses the application of a wide variety of methods and algorithms that extract rules and functions from large datasets. In our study, we used three different classifiers Naive Bayes, Random forest and J48. The **Naive Bayes** classifier, is based on the Bayesian theorem, and assumes that each predictor is conditionally independent of the other [26]. The algorithm for **Random forest** (RF), a form of multiple decision trees, was developed by Leo Breiman [27]. **J48**, a version of earlier algorithm (the very popular C4.5) developed by J. Ross Quinlan, builds decision trees from a set of labelled training data using the fact that each attribute of the data can be used to make a decision by splitting the data into smaller subsets [28].

Cost sensitivity was introduced by means of meta-learners. The two meta-learners employed in this study were *MetaCost* for J48 and *CostSensitiveClassifier* for Naive Bayes and Random Forest respectively [29].

### Model assessment

Standard ML statistical measures such as Accuracy, Sensitivity, Specificity, Balanced Classification Rate (BCR) and Receiver Operating Characteristic curve (ROC) were used to evaluate the performance of the classifiers. Accuracy is the percentage of predictions that are correct ((TP + TN)/(TP + TN + FP + FN)). Sensitivity is the percentage of positive labelled instances that are predicted as positive (TP/(TP + FN)). Specificity refers to percentage of negative labelled instances that are predicted as negative (TN/(TN + FP)). BCR is the average of sensitivity and specificity and enforces balance in the correct classification rate between two classes. A ROC curve is a graphical plot of True Positive rate vs. False Positive rate that illustrates a binary classifier’s performance by means of area under the curve (AUC).

### Maximum common substructure search

In order to identify potentially enriched substructures in the bioactive molecules, we employed a Maximum Common Substructure (MCS) based approach. We used a MCS based hierarchical clustering algorithm ‘LibMCS’ available from ChemAxon [30]. The minimal MCS size was empirically set to ’8’ atoms owing to the size and structural complexity of the molecules.

The molecular scaffolds thus generated as a result of MCS clustering were then used for similarity searching in active and inactive datasets using the *‘jcsearch’* algorithm available from ChemAxon [31]. The evaluation of substructures was done using the chi-square test. The p-value which is the probability value associated with chi-square was used to test the significance of enrichment. Using the vROCS (release 3.1.2) [32] we performed a molecular alignment of the selected scaffolds with molecules of active dataset and visualized the alignment in VIDA (4.1.1) [33] available from OpenEye Scientific Software, Inc. [34].

## Competing interests

The authors declare that they have no competing interests.

## Authors’ contributions

SJ and VP under the guidance of VS designed the study, carried out the work flow and performed the analysis. OSDDC was involved in regular discussions and supported the work. All authors contributed to manuscript writing, and have read and approved, the final manuscript.

## Supplementary Material

Additional file 1 List of descriptors calculated for the dataset. Click here for file
